# Prenatal Exposure to Environmental Phenols: Concentrations in Amniotic Fluid and Variability in Urinary Concentrations during Pregnancy

**DOI:** 10.1289/ehp.1206335

**Published:** 2013-08-13

**Authors:** Claire Philippat, Mary S. Wolff, Antonia M. Calafat, Xiaoyun Ye, Rebecca Bausell, Molly Meadows, Joanne Stone, Rémy Slama, Stephanie M. Engel

**Affiliations:** 1 Inserm, Institut Albert Bonniot (U823), Team of Environmental Epidemiology applied to Reproduction and Respiratory Health, Grenoble, France; 2 Grenoble University, Institut Albert Bonniot, Grenoble, France; 3 Department of Preventive Medicine, Mount Sinai School of Medicine, New York, New York, USA; 4 Centers for Disease Control and Prevention, Atlanta, Georgia, USA; 5 Columbia University College of Physicians and Surgeons, New York, New York, USA; 6 Department of Obstetrics, Gynecology, and Reproductive Sciences, Mount Sinai School of Medicine, New York, New York, USA; 7 Department of Epidemiology, Gillings School of Global Public Health, University of North Carolina at Chapel Hill, Chapel Hill, North Carolina, USA

## Abstract

Background: Maternal urinary biomarkers are often used to assess fetal exposure to phenols and their precursors. Their effectiveness as a measure of exposure in epidemiological studies depends on their variability during pregnancy and their ability to accurately predict fetal exposure.

Objectives: We assessed the relationship between urinary and amniotic fluid concentrations of nine environmental phenols, and the reproducibility of urinary concentrations, among pregnant women.

Methods: Seventy-one women referred for amniocentesis were included. Maternal urine was collected at the time of the amniocentesis appointment and on two subsequent occasions. Urine and amniotic fluid were analyzed for 2,4- and 2,5-dichlorophenols, bisphenol A, benzophenone-3, triclosan, and methyl-, ethyl-, propyl-, and butylparabens using online solid phase extraction–high performance liquid chromatography–isotope dilution tandem mass spectrometry.

Results: Only benzophenone-3 and propylparaben were detectable in more than half of the amniotic fluid samples; for these phenols, concentrations in amniotic fluid and maternal urine collected on the same day were positively correlated (ρ = 0.53 and 0.32, respectively). Other phenols were detected infrequently in amniotic fluid (e.g., bisphenol A was detected in only two samples). The intraclass correlation coefficients (ICCs) of urinary concentrations in samples from individual women ranged from 0.48 and 0.62 for all phenols except bisphenol A (ICC = 0.11).

Conclusion: Amniotic fluid detection frequencies for most phenols were low. The reproducibility of urine measures was poor for bisphenol A, but good for the other phenols. Although a single sample may provide a reasonable estimate of exposure for some phenols, collecting multiple urine samples during pregnancy is an option to reduce exposure measurement error in studies regarding the effects of phenol prenatal exposure on health.

Citation: Philippat C, Wolff MS, Calafat AM, Ye X, Bausell R, Meadows M, Stone J, Slama R, Engel SM. 2013. Prenatal exposure to environmental phenols: concentrations in amniotic fluid and variability in urinary concentrations during pregnancy. Environ Health Perspect 121:1225–1231; http://dx.doi.org/10.1289/ehp.1206335

## Introduction

Phenolic compounds are used in numerous consumer products such as cosmetics (parabens), sunscreens (benzophenone-3), antibacterial soaps (triclosan), and food packaging and polycarbonate plastics (bisphenol A). 2,5-Dichlorophenol is a metabolite of *p*-dichlorobenzene, a chemical used in mothballs and bathroom deodorizers. 2,4-Dichlorophenol is used primarily in the production of phenoxy acid herbicides (e.g., 2,4-diphenoxyacetic acid) and for the synthesis of pharmaceuticals and antiseptics. Exposure to these chemicals or their precursors is ubiquitous in the general population, including pregnant women (Casas et al. 2013; Wolff et al. 2008). Metabolites of phenols have also been detected in amniotic fluid (Edlow et al. 2012; Engel et al. 2006; Yamada et al. 2002) and cord blood (Chou et al. 2011; Fenichel et al. 2012), suggesting fetal exposure.

Certain environmental chemicals (e.g., phenols) are rapidly metabolized and excreted in urine after exposure. Thus, urinary concentrations are commonly used in epidemiological studies to estimate exposure to these chemicals. The extent to which concentrations in maternal urine reflect fetal exposure is uncertain. Alternative matrices, such as amniotic fluid, might provide a more accurate measure of direct fetal exposure. During pregnancy, the fetus swallows amniotic fluid, and fetal urine is one of the main sources of amniotic fluid in the second half of pregnancy (Modena and Fieni 2004; Underwood et al. 2005). Thus, concentrations measured in this matrix might be a relevant dosimeter of fetal exposure. In humans, bisphenol A (Edlow et al. 2012; Engel et al. 2006; Ikezuki et al. 2002; Yamada et al. 2002) and 2,5-dichlorophenol (Bradman et al. 2003) have been detected in amniotic fluid collected from pregnant women receiving amniocentesis as part of their routine prenatal care. However, to our knowledge, no published study has evaluated human amniotic fluid concentrations of other prevalent environmental phenols, nor has there been a comparison of phenol biomarker concentrations in amniotic fluid with corresponding concentrations in maternal urine.

There have been few studies of the health effects of prenatal exposures to phenols or their precursors, and all have relied on a relatively small number (1–3) of maternal urine samples collected during pregnancy to assess exposures (Philippat et al. 2012; Wolff et al. 2008). However, many phenols have short half-lives (< 1 day) in humans (Janjua et al. 2008; Teeguarden et al. 2011; Volkel et al. 2002), and exposures to these compounds are closely related to dietary intakes and episodic personal behaviors such as cosmetic use. Therefore, urinary concentrations are likely to vary throughout pregnancy. Variation in sample collection and processing may be an additional source of variability in phenol biomarker concentrations, and a statistical method to standardize measured concentrations for such factors (Mortamais et al. 2012) has been applied in epidemiological studies of associations between urinary biomarkers and health end points (Chevrier et al. 2012; Philippat et al. 2012). However, to our knowledge, the impact of standardizing biomarker concentrations on reproducibility measures has not been studied.

Our objectives were to assess the relationship between phenol biomarker concentrations measured in maternal urine and amniotic fluid collected on the same day, and to study the variability in urinary phenol concentrations over the course of pregnancy. A secondary aim was to assess the impact of collection conditions on the measured urine phenol concentrations, and determine whether standardization for these conditions improves reliability measures in phenol concentrations during pregnancy.

## Methods

*Study population*. The Study of Advanced Reproductive Age and Environmental Health (SARAEH) recruited 97 women who were referred for amniocentesis screening at the Mount Sinai Medical Center in New York, New York (USA). Recruitment occurred between 2005 and 2008. Study eligibility requirements were fluency in English or Spanish, maternal age between 18 and 40 years, singleton pregnancy, and intent to deliver at Mount Sinai Hospital. Women were approached for participation in the waiting area before their amniocentesis. Those who consented to participate in the study provided a spot urine sample immediately following their procedure. Two additional spot urine samples were collected from each participant later in their pregnancy, with collections planned at least 2 weeks apart, and the final sample collected after the 27th week of gestation. The present study comprises 71 participants who provided all three urine samples, including 69 for whom an amniotic fluid sample also was available. Following delivery, medical records were abstracted for information pertaining to fetal health.

This study was approved by the Mount Sinai Program for the Protection of Human Subjects, and all women gave informed consent to be part of the study. The involvement of the Centers for Disease Control and Prevention (CDC) laboratory was determined not to constitute engagement in human subjects research.

*Exposure assessment*. Following collection, amniotic fluid was delivered to the Mount Sinai Medical Center clinical cytogenetics laboratory for standard clinical care processing, which includes centrifugation and cell separation. Before being released for research purposes, amniotic fluid supernatant was stored in polypropylene containers at –20°C (time range, 0–38 weeks). Once the samples were released, SARAEH research personnel thawed the amniotic fluid overnight at 4°C, and then aliquoted the supernatant into 2-mL cryovials for storage at –80°C. Urine and amniotic fluid samples were shipped overnight on dry ice to the CDC for measurements of total (free plus conjugated) concentrations of 2,4- and 2,5-dichlorophenols, bisphenol A, benzophenone-3, triclosan, and methyl-, ethyl-, propyl-, and butylparabens by using online solid phase extraction–high performance liquid chromatography–isotope dilution tandem mass spectrometry (Ye et al. 2005). Specific gravity of urine was measured using a handheld refractometer in each thawed aliquot before shipment to the CDC, except for 11 urine samples that had specific gravity measured using an Atago PAL-10S refractometer (Atago, Bellevue, WA, USA) at the CDC. Urinary creatinine concentration was measured at CDC by an enzymatic reaction using a Roche Hitachi 912 chemistry analyzer (Hitachi, Pleasanton, CA, USA).

*Statistical analyses*. Analyses were conducted on natural-log (ln)–transformed concentrations. Amniotic fluid concentrations below the limit of detection (LOD) were replaced with the instrument reading values for phenols detected in at least 50% of the samples. Instrument reading values were not available for urine assays; therefore, urine phenol concentrations below the LOD were replaced with the LOD/_√_^–^2. The following formula was used to correct urinary concentrations for specific gravity: C_SG_ = C × [(SG_mean_ – 1)/(SG – 1)], where C_SG_ is the specific gravity–corrected biomarker concentration, SG_mean_ is the specific gravity arithmetic mean in our population, and C is the measured biomarker concentration. Creatinine-corrected concentrations (micrograms per gram) were calculated by dividing the phenol concentrations (micrograms per liter) by the creatinine concentration (milligrams per deciliter) and multiplying by 100.

*Relation between maternal urine and amniotic fluid biomarker concentrations*. We compared amniotic fluid and urinary biomarker concentrations collected on the same day, and computed ratios of uncorrected urine to amniotic fluid concentrations. For benzophenone-3 and propylparaben, the two phenols detected in > 50% of the amniotic fluid samples, we computed Spearman correlation coefficients comparing concentrations in amniotic fluid and urinary samples (uncorrected and specific gravity corrected) collected on the same day. To explore the possible predictors of benzophenone-3 and propylparaben concentrations in amniotic fluid, we performed Tobit regression models for a left-censored dependent variable (Lubin et al. 2004). We regressed amniotic fluid biomarker concentrations on maternal urinary concentrations in samples collected on the same day, fetal sex, gestational age at amniocentesis (< 17, 17–18.9, ≥ 19 gestational weeks), maternal age (< 31, 31–35.9, ≥ 36 years), maternal prepregnancy body mass index (BMI; < 25 or ≥ 25 kg/m^2^), race/ethnicity (white non-Hispanic or other), pregnancy complications related to placental function (including preeclampsia, placenta previa, small placenta, oligohydroamnios, and chorioamnionitis), and the time between amniotic fluid collection and processing (≤ 4, 4 to 16, 16–20, or > 20 weeks). Models were simultaneously adjusted for all of these factors. We also performed sensitivity analyses excluding four women with abnormal amniotic fluid conditions (oligohydramnios or polyhydramnios).

*Variability in urinary concentrations*. We evaluated variability in measures of dilution in spot urine samples across pregnancy by plotting urinary creatinine concentrations and urine specific gravity as a function of gestational age. To assess variability in phenol concentrations across pregnancy, we computed intraclass correlation coefficients (ICCs) between concentrations measured in the three spot urine samples using random intercept linear mixed models (Rabe-Hesketh 2008). The ICC is the ratio of the between-women variability to the total variability (between- plus within-woman variability). We also computed Spearman correlations between pairs of phenol concentrations measured in the three spot urine samples from each woman. We classified the comparability of samples based on ICCs and Spearman correlation coefficients according to the following general guidelines: < 0.4, weak; 0.4–0.6, moderate; > 0.6, good. We performed analyses of urinary concentration variability using uncorrected, specific gravity–corrected, and creatinine-corrected concentrations. Creatinine concentrations were missing for 11 of the first spot urine samples. Thus, analyses based on creatinine-corrected concentrations were restricted to 60 women with complete creatinine and environmental phenol biomarker data.

In a secondary analysis, we computed Spearman correlations among pairs of urine samples collected within specific time intervals: < 4 weeks apart, 4–6 weeks apart, 6–10 weeks apart, 10–12 weeks apart, 12–14 weeks apart, 14–16 weeks apart, and > 16 weeks apart. Samples were not independent within a given category because multiple samples from an individual woman could be included in a single category if they were collected at equally spaced intervals.

Finally, we estimated associations between urinary phenol biomarker concentrations and collection conditions (gestational age at collection; hour, day, and season of sampling; urine specific gravity) using a random intercept linear mixed model simultaneously adjusted for each collection condition along with BMI, maternal age, year of collection, maternal education, and maternal race/ethnicity (modeled as indicated previously) (Mortamais et al. 2012). We used the measured urinary biomarker concentrations and the estimated effects of collection conditions on the measured urine concentrations (for conditions that predicted urine concentrations with *p* < 0. 2) to derive standardized concentrations—concentrations that would have been observed if all samples had been collected under the same conditions (Mortamais et al. 2012). We estimated ICCs based on concentrations standardized for collection conditions to determine whether standardization improved reliability across repeated spot urine samples.

All analyses were performed using STATA/SE, version 12 (StataCorp, College Station, TX, USA).

## Results

*Study population and exposure*. Women averaged 35.5 years old and were mostly white (72%), and 55% attended graduate school (Table 1). The three urine samples were collected (mean ± SD) at 17.6 ± 1.6, 22.8 ± 3.5, and 32.8 ± 3.1 gestational weeks, respectively. On average, the first two urine samples were collected 5 ± 2.8 weeks apart, and the second and third were collected 10 ± 3.9 weeks apart. Overall, the first urine sample was collected later in the day than the other samples, and the number of women with urine samples collected before 1000 hours was 1, 15, and 16 for the first, second, and third samples, respectively. Except for ethylparaben, median values of measured phenol concentrations (uncorrected for urine dilution), creatinine concentrations, and specific gravity were lowest in the first urine samples (Table 2). Detection frequencies of environmental phenols in urine were high: 2,5-Dichlorophenol, benzophenone-3, methylparaben, and propylparaben were detected in at least 99% of the samples, and detection frequencies for the other phenols ranged between 59% and 94% (Table 2). Detection frequencies in amniotic fluid were much lower: Only benzophenone-3 and propylparaben were detectable in at least half of the samples (61% and 58%, respectively). Bisphenol A was detected in two amniotic fluid samples only (at a concentration equal to the LOD of 0.4 μg/L), but was detected in 62% of the urine samples collected on the same day (Table 2).

**Table 1:** Characteristics of 71 women with three urine measurements during pregnancy: SARAEH study, 2005–2008.

Characteristic	*n *(%)
Maternal age (years)
25–29.9	5 (7)
30–34.9	21 (30)
35–40.9	45 (63)
Race/ethnicity
White	51 (72)
Black	5 (7)
Hispanic	11 (15)
Asian	3 (4)
American Indian	1 (1)
Pre­pregnancy BMI (kg/m^2^)
17.8–18.4	3 (4)
18.5–24.9	53 (75)
25–41	15 (21)
Maternal education
High school	9 (13)
Any college	23 (32)
Graduate school, graduate degree	39 (55)
Conception method
Reproductive techniques	3 (4)
Natural	68 (96)
Amniotic fluid sampling reason
Advanced maternal age	49 (69)
Abnormal genetic screen	10 (14)
Elective	11 (15)
Other	1 (1)

**Table 2:** Distribution of phenol concentrations (μg/L) measured in amniotic fluid and in urine collected at three time periods during pregnancy among women of the SARAEH study, 2005–2008.

Analyte	LOD	Urine sample (*n* = 71)^*a*^												Amniotic fluid (*n* = 69)^*b*^				Ratio of first urine sample:amniotic fluid		
		First				Second				Third										
		% > LOD	5th	50th	95th	% > LOD	5th	50th	95th	% > LOD	5th	50th	95th	% > LOD	5th	50th	95th	5th	50th	95th
Phenols (μg/L)
2,4‑Dichlorophenol^*c*^	0.2	68	< LOD	0.6	5.7	94	< LOD	1.1	8.3	93	< LOD	1.0	14	1^*c*^	< LOD	< LOD	LOD	1.4	1.4	9
2,5‑Dichlorophenol	0.2	100	0.8	7.5	178	100	1.8	12.8	364	100	1.7	14.2	466	16	< LOD	LOD	5.2	3.5	297	1,756
Bisphenol A	0.4	62	< LOD	0.6	7.0	92	< LOD	1.8	9.5	82	< LOD	1.2	5.0	3	< LOD	< LOD	LOD	1.4	12	85
Benzophenone‑3	0.4	100	4.1	53.5	2,220	100	5.7	77	6,740	100	8.9	76.1	3,180	61	< LOD	0.8	15.7	8.0	110	1,648
Triclosan	2.3	79	< LOD	6.5	789	75	< LOD	15.4	734	80	< LOD	16.2	514	6	< LOD	< LOD	19.4	1.4	16	354
Methylparaben	1.0	100	9.6	100.0	985	100	19	272	1,670	100	9.7	156	1,830	42	< LOD	< LOD	3.3	23.0	196	827
Ethylparaben	1.0	59	< LOD	2.8	58	65	< LOD	4.5	347	59	< LOD	1.7	144	0	< LOD	< LOD	< LOD	3.5	30	304
Propylparaben	0.2	100	0.5	28.7	424	100	1.6	45.6	531	99	0.8	36.5	589	58	< LOD	0.3	1.4	7.0	116	2,130
Butylparaben	0.2	70	< LOD	1.5	26	85	< LOD	1.9	56.3	73	< LOD	1.7	58.2	6	< LOD	< LOD	0.3	1.4	22	179
Markers of urine dilution
Creatinine (mg/dL)^*d*^	3.5	100	13.7	44.1	256	100	15.3	86.8	221	100	16	90.9	175
Specific gravity			1.003	1.008	1.026		1.003	1.02	1.03		1.004	1.015	1.026
5th, 50th, and 95th are percentiles. ^***a***^Concentrations not corrected for urine dilution; concentrations < LOD were replaced by LOD/√–2. ^***b***^Concentrations < LOD were replaced by instrumental reading values. ^***c***^2,4-DCP was assessed in only 11 amniotic fluid samples. ^***d***^*n *= 60; creatinine concentration was missing for 11 urine samples from the first sampling.																				

*Relation between maternal urine and amniotic fluid biomarker concentrations*. Among women with no abnormal amniotic fluid condition (i.e., excluding four participants with oligohydramnios or polyhydramnios), ratios of concentrations in the first urine sample (not corrected for urine dilution) to concentrations in amniotic fluid were > 1 (Table 2). However, benzophenone-3 and butylparaben concentrations were higher in amniotic fluid than in the corresponding urine sample for one woman with oligohydramnios (urine:amniotic fluid ratios of 0.2 for benzophenone-3 and 0.7 for butylparaben).

**Figure 1:**
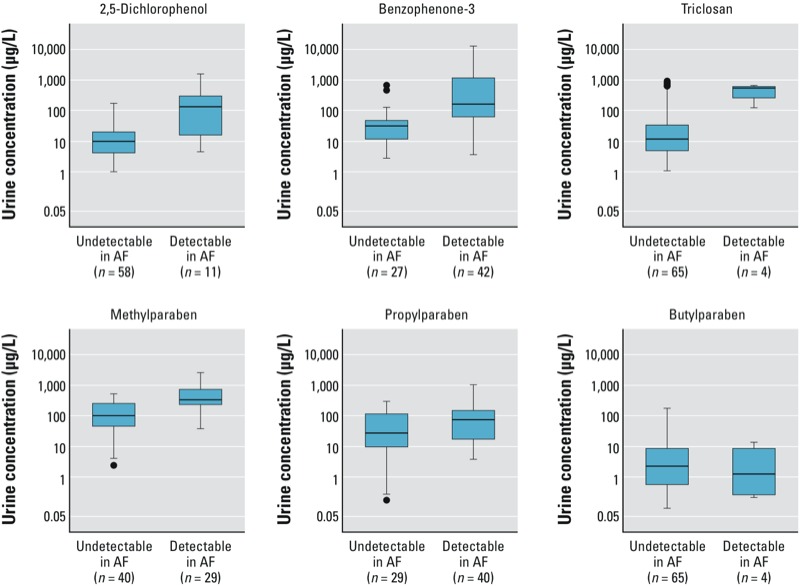
Box plots of the distribution of specific gravity–corrected urinary phenol concentrations according to undetected or detectable levels of phenol metabolites in amniotic fluid (AF) collected on the same day. In general, urinary concentrations were higher in women with detectable amniotic fluid concentrations. Boxes represent the 25th and 75th percentiles, lines inside the boxes are medians, whiskers represent values within the 1.5 IQR of the 25th and 75th percentiles, and circles represent outliers. Distributions are not shown for bisphenol A, 2,4-dichlorophenol, or ethylparaben because they were detected in only 2, 1, and 0 amniotic fluid samples, respectively.

Except for butylparaben, which was detected in only four amniotic fluid samples, median urinary concentrations of phenol biomarkers (with or without correction for dilution) were higher in women with detectable amniotic fluid concentrations (Figure 1). The correlation between amniotic fluid and urinary concentrations (uncorrected for urine dilution) was moderate for benzophenone-3 (ρ = 0.53) and weak for propylparaben (ρ = 0.32). Correction for urinary dilution had little effect on these correlations (data not shown).

After adjustment (for storage time, gestational age at sampling, maternal age, BMI, race/ethnicity, pregnancy complications, and child’s sex), ln-amniotic fluid concentrations increased by 0.52 (95% CI: 0.3, 0.7) and 0.36 (95% CI: 0.2, 0.5) with a 1-unit increase in the ln-transformed specific gravity–corrected urinary concentration of benzophenone-3 and propylparaben, respectively (Table 3). Including urine specific gravity as a covariate instead of using phenol concentrations corrected for specific gravity did not change the results (data not shown). After exclusion of the four women with abnormal amniotic fluid conditions (oligohydramnios and polyhydramnios), urine and amniotic fluid biomarker concentrations were still positively associated (data not shown). There was a limited range of gestational ages, and no clear associations between gestational age at urine collection and amniotic fluid concentrations. Maternal age (*p*-trend = 0.09) and nonwhite race/ethnicity (*p* = 0.16) were negatively associated with amniotic concentrations of benzophenone-3. Propylparaben and benzophenone-3 concentrations in amniotic fluid were lower for female fetuses than for male fetuses (β = –0.56; 95% CI: –1.2, 0.1 and β = –0.37; 95% CI: –1.2, 0.4, respectively) (Table 3).

**Table 3:** Adjusted associations of ln-transformed amniotic fluid concentrations of benzophenone-3 and propylparaben^*a*^ with potential predictors among 68 pregnant women from the SARAEH study, 2005–2008.

Characteristic	*n*	Change in ln-BP3 concentration (μg/L) β (95% CI)	Estimated BP3 concentration (GM, μg/L)	*p*-Trend^*b*^	Change in ln-PP concentration (μg/L) β (95% CI)	Estimated PP concentration (GM, μg/L)	*p*-Trend^*b*^
Urine concentration (ln-unit, μg/L)^*c*^		0.52 (0.3, 0.7)			0.36 (0.2, 0.5)
Time of amniotic fluid storage at –20°C (weeks)	0.35	0.35
≤ 4	17	Reference	0.71		Reference	0.20
4–16	12	–0.28 (–1.6, 1.0)	0.54		0.93 (0.0, 1.9)	0.50
16–20	14	–0.39 (–1.7, 0.9)	0.48		0.02 (–0.9, 1.0)	0.20
> 20	27	–0.61 (–1.9, 0.7)	0.39		–0.34 (–1.3, 0.6)	0.14
Gestational age at sampling (weeks)	0.71	0.49
15–16.9	31	Reference	0.51		Reference	0.20
17–18.9	26	0.11 (–0.7, 0.9)	0.57		–0.18 (–0.8, 0.4)	0.17
19–23	11	–0.35 (–1.8, 1.1)	0.36		0.55 (–0.4, 1.5)	0.35
Maternal age (years)	0.09	0.56
< 31	10	Reference	1.03		Reference	0.18
31–35.9	24	–0.59 (–1.8, 0.7)	0.58		0.06 (–0.9, 1.0)	0.20
36–40	34	–1.04 (–2.3, 0.2)	0.37		0.20 (–0.7, 1.1)	0.23
BMI	0.67	0.34
< 25	53	Reference	0.53		Reference	0.23
≥ 25	15	–0.23 (–1.3, 0.9)	0.42		–0.39 (–1.2, 0.4)	0.15
Race/ethnicity
White	48	Reference	0.63		Reference	0.20
Nonwhite	20	–0.77 (–1.9, 0.3)	0.29		0.16 (–0.6, 0.9)	0.23
Pregnancy complications related to placenta^*d*^
No	58	Reference	0.50		Reference	0.23
Yes	10	–0.05 (–1.3, 1.2)	0.48		–0.79 (–1.7, 0.1)	0.11
Fetal sex
Male	36	Reference	0.59		Reference	0.27
Female	32	–0.37 (–1.2, 0.4)	0.41		–0.56 (–1.2; 0.1)	0.15
Abbreviations: AF, amniotic fluid; BP3, benzophenone-3; PP, propylparaben; GM, geometric mean. ^***a***^Phenols detected in at least 50% of the amniotic fluid samples. ^***b***^*p*-Trend was estimated using categorical variables whose values corresponded to the category-specific median value (Rothman et al. 2008). ^***c***^Corrected for specific gravity. ^***d***^Including oligohydroamnios, choriamniotis, small placenta, placenta praevia and preeclampsia. This analysis was restricted to 68 pregnant women because the fetal sex was missing for one woman.							

*Variability in urinary concentrations*. We observed similar variations in urinary specific gravity and creatinine concentrations throughout pregnancy (see Supplemental Material, Figure S1), and both measures of urinary dilution were highly correlated (Spearman correlation coefficient = 0.93). ICCs (Table 4) and Spearman correlations (see Supplemental Material, Table S1) were similar for urine concentrations corrected for creatinine or specific gravity and, except for ethylparaben and bisphenol A, were always higher than corresponding estimates for concentrations that were not corrected for dilution. Given that, and because creatinine concentrations are missing for 11 urine samples, we discuss only specific gravity–corrected estimates going forward.

**Table 4:** ICCs for phenol biomarker concentrations measured in urine samples collected at several times during pregnancy: SARAEH study, 2005–2008.

Analyte	Specific gravity–corrected concentration^*a*^	Creatinine-corrected concentration^*b*^	Uncorrected concentration^*a*^	Concentration standardized for collection conditions^*a*^^,^^*c*^
2,4‑Dichlorophenol	0.60	0.48	0.47	0.60
2,5‑Dichlorophenol	0.61	0.62	0.52	0.60
Bisphenol A	0.11	0.14	0.23	0.15
Benzophenone‑3	0.62	0.70	0.57	0.61
Triclosan	0.58	0.61	0.56	0.60
Methylparaben	0.61	0.61	0.52	0.62
Ethylparaben	0.48	0.44	0.48	0.48
Propylparaben	0.55	0.54	0.51	0.54
Butylparaben	0.56	0.64	0.54	0.61
^***a***^*n *= 213 samples from 71 women. ^***b***^*n *= 180 samples from 60 women with creatinine measured in all three urine samples. ^***c***^All concentrations were standardized for specific gravity. Bisphenol A concentration was additionally standardized for gestational age at sampling and sampling hour. Butylparaben, triclosan, and 2,4-dichlorophenol were additionally standardized for hour of sampling. Benzophone-3 and methylparaben were additionally standardized for hour and season of sampling, propylparaben for season of sampling, and ethylparaben for day of sampling.				

ICCs were moderate (0.4–0.6) to good (> 0.6), and of comparable magnitude, for 2,4- and 2,5-dichlorophenol, benzophenone-3, triclosan, and methyl-, ethyl- propyl-, and butylparabens (Table 4), with similar patterns for Spearman correlation coefficients, which ranged from 0.41 to 0.77 (see Supplemental Material, Table S1). In contrast, the ICC for bisphenol A was only 0.11 (Table 4). Although collections of spot urines were planned to occur at least 2 weeks apart, four women had an interval < 2 weeks between successive collections. However, excluding these women did not change our results (data not shown).

Correlations between urinary concentrations measured within different time intervals are shown in Supplemental Material, Table S2. Our expectation was that correlations would be higher for samples collected with shorter intervening time intervals, and longer for samples collected with longer intervening time intervals. As expected, for 2,4- and 2,5-dichlorophenol, ethylparaben, and benzophenone-3, but not for the other phenols, we observed lower coefficients overall (i.e., less reliability) for samples with longer time intervals between collections. Correlations for bisphenol A were weak, regardless of the time between sample collections.

Specific gravity was positively associated with all urinary phenol biomarker concentrations (*p*-values ≤ 0.1) (see Supplemental Material, Table S3). Similar associations were observed with creatinine concentrations (data not shown). We observed statistically significant (*p* < 0.05) heterogeneity in 2,4-dichlorophenol, benzophenone-3, triclosan, and butylparaben concentrations according to hour of sampling, and in bisphenol A concentration according to gestational age. Benzophenone-3 concentrations were significantly higher in samples collected in spring and summer (adjusted geometric mean, 110 μg/L) than in samples collected in fall and winter (adjusted geometric mean, 69 μg/L). Race/ethnicity was associated with benzophenone-3 concentration in urine, which was higher among white non-Hispanic than among nonwhite women (β = –1.2, 95% CI: –2.1, –0.3). Urinary concentrations standardized for collection conditions (Mortamais et al. 2012) and urinary concentrations corrected for specific gravity were strongly correlated (Spearman correlation coefficients > 0.96). The use of concentrations standardized for collection conditions did not substantially alter ICCs for spot urine collections from ICCs for concentrations that were uncorrected or corrected for dilution (Table 4).

## Discussion

In our population of pregnant women referred for amniocentesis, the detection frequencies of phenol biomarkers in amniotic fluid were low, with only two phenols detected in ≥ 50% of the samples. Except for bisphenol A (ICC = 0.11), the reproducibility of urine phenol biomarker concentrations in urine collected at three times during pregnancy was fairly good (ICCs of 0.48–0.62 for specific gravity–corrected concentrations).

*Phenol concentrations in maternal urine*. Urine collected on the day of the amniocentesis tended to be more dilute than urine collected later in pregnancy; however, no specific instructions (with respect to hydration) or procedures (e.g., use of intravenous therapy) that would affect urinary dilution were given before or during the amniocentesis. Methyl- and propylparabens are the most commonly used parabens in food and cosmetics (Soni et al. 2005), and, as expected, urine concentrations of these phenols were higher than those observed for ethyl- and butylparabens. Triclosan and bisphenol A concentrations were similar, and 2,5-dichlorophenol concentrations were lower than those reported in a previous mother–child cohort study conducted at the Mount Sinai Medical Center between 1998 and 2002, which included predominantly Hispanic and African-American participants [medians were 11 μg/L for triclosan, 1.3 μg/L for bisphenol A, and 53 μg/L for 2,5-dichlorophenol (Wolff et al. 2008), compared with medians of 6.5–16.2, 0.6–1.8, and 7.5–14.2 μg/L, respectively, in our study population (ranges of median concentrations for the three time points)]. Urinary concentrations of parabens were not reported in the study published by Wolff et al. (2008). Median urinary concentrations of benzophenone-3 in our study population (54–77 μg/L) were considerably higher than those reported in the aforementioned study (median of 7.5 μg/L). It is likely that this partly reflects the different racial/ethnic composition of these two Mount Sinai studies, because exposure to benzophenone-3 in sunscreens (Summers et al. 2011) would be more common in the predominantly white participants. In our study, being non-Hispanic white has been associated with higher benzophenone-3 concentrations in urine and in amniotic fluid.

*Phenol concentrations in amniotic fluid*. Although the presence of phenol biomarkers in amniotic fluid highlights the potential for direct fetal exposure, the low detection frequency observed for most of these compounds may be suggestive of low transfer and/or rapid clearance of the phenols in amniotic fluid. That being said, we cannot exclude the possibility that the low detection of phenol biomarkers in amniotic fluid was the result of processing and/or storage of samples in the clinical laboratory before being released for research purposes. Ye et al. (2007) reported good stability of total phenol concentrations in urine stored at –70°C, but, to our knowledge, there are no published data regarding the impact of storage at –20°C or the stability of biomarker concentrations in amniotic fluid.

We detected bisphenol A in only two amniotic fluid samples. Low concentrations of bisphenol A in amniotic fluid collected during pregnancy and at birth have been previously reported (medians ranged from 0.26 to 1.1 μg/L) (Edlow et al. 2012; Engel et al. 2006; Ikezuki et al. 2002; Yamada et al. 2002). In an *ex vivo* experimental system, where seven human placentas were in contact with a perfusate enriched in bisphenol A (10 μg/L), Balakrishnan et al. (2010) observed rapid transfer of this phenol across the placenta; however, the concentration used in the experimental study was higher than those typically observed in the urine of pregnant women from the general population (Woodruff et al. 2011). Bradman et al. (2003) reported a higher geometric mean of 2,5-dichlorophenol concentrations (0.39 μg/L) in second-trimester amniotic ﬂuid samples from 20 women referred for amniocentesis at Children’s Hospital Central California in Madera, California, than we observed in our population (geometric mean = 0.29 μg/L). To our knowledge, no previous study has examined amniotic fluid concentrations of benzophenone-3, triclosan, or parabens in humans.

*Predictors of amniotic fluid concentrations*. Women with the highest concentrations of phenols in urine were more likely to have detectable concentrations of environmental phenols in amniotic fluid. Because of the high percentage of undetectable concentrations, we were able to evaluate predictors of amniotic fluid concentrations only for benzophenone-3 and propylparaben, and for these substances, maternal urine concentration was found to be a strong predictor of amniotic fluid concentration. There have been few comparisons of maternal and fetal exposure for phenolic compounds (Ikezuki et al. 2002; Yamada et al. 2002). Ikezuki et al. (2002) compared bisphenol A concentration in maternal serum (*n* = 37 samples) and amniotic fluid (*n* = 32 samples collected at 15–18 weeks), and reported amniotic fluid biomarker concentrations (arithmetic mean, 8.3 μg/L) about five times higher than in maternal serum (arithmetic mean = 1.5 μg/L). However, mean concentration of 8.3 μg/L is substantially higher than amniotic fluid concentrations reported by others (median values from 0.26 to 1.1 μg/L) (Edlow et al. 2012; Engel et al. 2006; Yamada et al. 2002), raising concerns regarding potential external contamination from environmental sources of free bisphenol A.

We found no evidence of an association between gestational age at collection and benzophenone-3 or propylparaben amniotic fluid concentrations. However, the range of gestational ages at amniocentesis (15–23 gestational weeks) was limited in our study population. Two previous studies reported higher bisphenol A concentrations in amniotic fluid collected in the second trimester than in samples collected later in pregnancy. However, in these studies, the samples collected in the second and third trimesters were from different women who may have had different levels of exposure (Edlow et al. 2012; Ikezuki et al. 2002). In our study population, the concentration of propylparaben in amniotic fluid was lower in female fetuses compared with male fetuses. This difference may have been attributable to chance, or could indicate sex differences in fetal metabolism or excretion.

*Variability in urinary concentrations*. The poor reproducibility of bisphenol A concentrations measured in serial spot urine samples collected during pregnancy is consistent with previous reports (Braun et al. 2011, 2012), and taken together, these findings suggest that substantial exposure misclassification might occur in epidemiological studies when prenatal bisphenol A exposure is classified using a single spot urine sample. Exposures to the eight other phenols or their precursors, which are found in sunscreens, cosmetics, and indoor deodorizers, were more consistent throughout pregnancy in our study population and had a higher degree of reproducibility in urinary concentrations, with ICCs of 0.5–0.6. Although a correlation of this magnitude is greater than reported for many other rapidly metabolized compounds, it may still result in bias due to exposure misclassification if the putative window of susceptibility is distant from the sample collection period. In a study relying on repeated measurements in indoor dust, Whitehead et al. (2012) reported a 40% attenuation of the effect estimates when only one measurement per household, rather than the mean of the repeated measurements per household, was used to assess exposure to chemicals with ICC of about 0.6. To limit the effects of exposure misclassification, studies of the effects of phenol prenatal exposure on health should try to collect multiple urine samples during pregnancy.

Two previous studies (Meeker et al. 2013; Smith et al. 2012) among pregnant women reported ICCs for methyl-, propyl-, and butylparabens (ICCs < 0.5) that were low relative to ICCs in our population; the authors did not measure ethylparaben concentration. Meeker et al. (2013) also reported lower ICCs for triclosan and 2,4- and 2,5-dichlorophenol (0.47, 0.38, and 0.49, respectively), but their ICC for benzophenone-3 (0.62) was the same as in our study population. Differences may be attributable to differences in exposure patterns between study populations, or to differences in the time between consecutive urinary collections. For example, urine samples were collected at gestational weeks 5.8, 20.6, and 33.5 on average in the Smith et al. study (2012), compared with 17.6, 22.8, and 32.8 weeks in our study.

Correcting for urine dilution improved the temporal reproducibility in urinary concentration for most of the phenols that we evaluated. Urinary creatinine and specific gravity followed similar patterns throughout pregnancy, and the estimates of urinary concentration reliability (correlations, ICCs) were similar for both methods. These findings suggest that either could be used to correct for urine dilution in pregnant women.

Because benzophenone-3 is commonly used in sunscreen for its sun-blocking properties (Calafat et al. 2008), exposure is more likely to be high during the summer months than the rest of the year, consistent with the seasonal variability in benzophenone-3 concentrations in our population (adjusted geometric means of 110 μg/L in April–September compared with 69 μg/L for samples collected October–March). Associations between hour of collection and benzophenone-3, triclosan, butylparaben, and 2,4-dichlorophenol urinary concentrations suggest that exposures changed considerably throughout the day. We sought to remove the variability in phenol urinary concentrations due to heterogeneity in collection conditions by implementing the two-step standardization method proposed by Mortamais et al. (2012). However, ICCs obtained with the standardized concentrations were close to those obtained from the unstandardized concentrations, suggesting that study-related variability in sample collection conditions did not strongly influence intrasubject differences in biomarker levels.

*Study strengths and limitations*. Our study is the first to report phenol concentrations in amniotic fluid and maternal urine collected on the same day. In addition, we examined variability in phenol biomarker concentrations during the prenatal period for a diverse set of environmental phenols, including some that have not been widely studied (triclosan, benzophenone-3, and dichlorophenols). Limitations include the relatively small sample size and the low detection frequency of phenols in amniotic fluid. Additionally, although we made no exclusions on the basis of race/ethnicity, most of our study population was white. Because there is some racial/ethnic variability in exposure patterns, our results may not fully represent variability in the general population.

To better understand the link between maternal and fetal exposures, larger studies with more diverse populations are needed. Collecting more urine samples may improve the characterization of the urine concentration variability during pregnancy.

## Conclusions

Our estimates of the reproducibility of benzophenone-3, triclosan, 2,4- and 2,5-dichlorophenols, and methyl-, ethyl-, propyl-, and butylparaben urinary concentrations over the course of the pregnancy suggest that more than one urine sample is needed to reasonably represent the individual’s exposure across pregnancy to these compounds or their precursors. As previously reported (Braun et al. 2011, 2012), we observed low reproducibility in bisphenol A urinary concentration among pregnant women, with little evidence of secondary fetal exposure to bisphenol A via amniotic fluid. Given the infrequent detection and much lower concentrations of most biomarkers in amniotic fluid than in urine, our results suggest that amniotic fluid may not be a suitable matrix for assessing fetal exposure to nonpersistent phenols using the currently available detection techniques.

## Supplemental Material

(426 KB) PDF
