# Supplementary material for: Prenatal Exposure to Environmental Phenols: Concentrations in Amniotic Fluid and Variability in Urinary Concentrations during Pregnancy
Source: Environ Health Perspect. 2013 Aug 13;121(10):1225–31. doi: 10.1289/ehp.1206335 (PMC3801458; doi:10.1289/ehp.1206335)
Supplement: (426 KB) PDF [file ehp.1206335.s001.pdf]

Supplemental Material

Prenatal Exposure to Environmental Phenols: Concentrations in Amniotic Fluid and Variability in Urinary Concentrations during Pregnancy

Claire Philippat, Mary S. Wolff, Antonia M. Calafat, Xiaoyun Ye, Rebecca Bausel, Molly Meadows, Joanne Stone, Rémy Slama, and Stephanie M. Engel

Table of Contents

Supplemental Material, Figure S1: Mean and 95% confidence intervals for urine specific gravity (A) and urine creatinine concentration (B) by gestational age, Study of Advanced Reproductive Age and Environmental Health, 2005 to 2008…………………..…………………….... 2

Supplemental Material, Table S1: Spearman correlation coefficients between pairs of ln-transformed phenol concentrations from the first, second and third samplings (specific gravity corrected, creatinine corrected, and uncorrected for urine dilution), Study of Advanced Reproductive Age and Environmental Health, 2005 to 2008…..........................3

Supplemental Material, Table S2: Spearman correlation coefficients between urine samples collected at specific time intervals using specific gravity corrected, creatinine corrected, and uncorrected phenol concentrations, Study of Advanced Reproductive Age and Environmental Health, 2005 to 2008……………………………………………………. 4

Supplemental Material, Table S3: Adjusted geometric mean of urinary phenol concentrations according to collection conditions among 213 urine samples from the 71 women of Study of Advanced Reproductive Age and Environmental Health, 2005 to 2008………………... 5

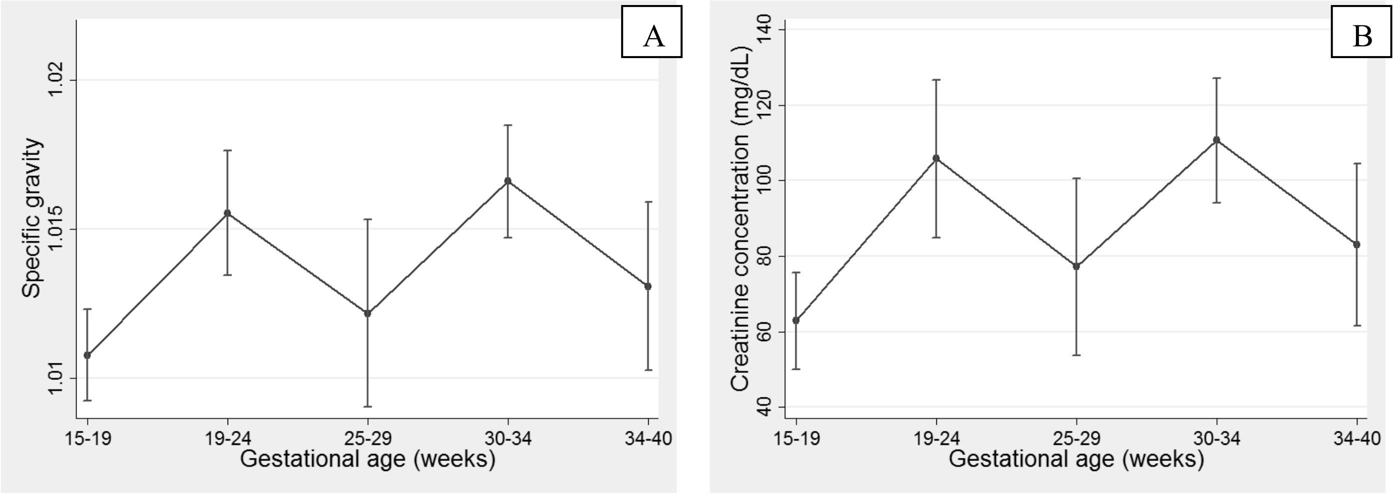

Supplemental Material, Figure S1: Mean and 95% confidence intervals for urine specific gravity (A) and urine creatinine concentration (B) by gestational age, Study of Advanced Reproductive Age and Environmental Health, 2005 to 2008. n = 213 urine samples for specific gravity and 202 for creatinine (creatinine concentrations were missing for 11 of the first spot urine samples).

Supplemental Material, Table S1: Spearman correlation coefficients between pairs of ln-transformed phenol concentrations from the first, second and third samplings (specific gravity corrected, creatinine corrected, and uncorrected for urine dilution), Study of Advanced Reproductive Age and Environmental Health, 2005 to 2008.

		2,4-dichlorophenol		2,5-dichlorophenol		Bisphenol A		Benzophenone-3		Triclosan		Methylparaben		Ethylparaben		Propylparaben		Butylparaben	
Sampling		1st	2nd	1st	2nd	1st	2nd	1st	2nd	1st	2nd	1st	2nd	1st	2nd	1st	2nd	1st	2nd
Specific gravity corrected concentrations (n = 71 women, 213 samples)																			
	2nd	0.70		0.75		0.1		0.77		0.57		0.53		0.51		0.48		0.59	
	3rd	0.54	0.62	0.56	0.63	0.26	0.18	0.53	0.64	0.49	0.61	0.63	0.73	0.41	0.53	0.53	0.54	0.49	0.61
Creatinine corrected concentrations (n = 60 women, 180 samples) [a]																			
	2nd	0.74		0.84		0.15		0.86		0.60		0.55		0.42		0.47		0.61	
	3rd	0.41	0.56	0.58	0.63	0.39	0.07	0.65	0.70	0.57	0.57	0.64	0.68	0.40	0.44	0.57	0.55	0.61	0.71
Crude concentrations (n = 71 women, 213 samples)																			
	2nd	0.59		0.70		0.25		0.68		0.59		0.47		0.48		0.45		0.59	
	3rd	0.35	0.56	0.43	0.59	0.42	0.28	0.47	0.60	0.53	0.58	0.58	0.58	0.48	0.47	0.56	0.50	0.52	0.62

[a] Creatinine concentrations were missing for 11 of the first spot urine samples.

Supplemental Material, Table S2: Spearman correlation coefficients between urine samples collected at specific time intervals using specific gravity corrected, creatinine corrected, and uncorrected phenol concentrations, Study of Advanced Reproductive Age and Environmental Health, 2005 to 2008.

	Time interval between samples (weeks)						
Analytes	<4	4 to 6	6 to 10	10 to 12	12 to 14	14 to 16	≥ 16
Specific gravity corrected concentrations							
No of sample pairs	32	32	30	36	24	29	30
2,4-dichlorophenol	0.79	0.71	0.70	0.67	0.69	0.49	0.29
2,5-dichlorophenol	0.83	0.71	0.70	0.61	0.59	0.71	0.40
Bisphenol A	0.29	-0.10	0.34	0.25	0.43	0.38	-0.32
Benzophenone-3	0.80	0.74	0.59	0.7	0.75	0.58	0.29
Triclosan	0.57	0.70	0.71	0.46	0.70	0.28	0.35
Methyl paraben	0.59	0.56	0.61	0.58	0.70	0.61	0.63
Ethyl paraben	0.58	0.45	0.44	0.49	0.48	0.48	0.26
Propyl paraben	0.51	0.43	0.64	0.37	0.38	0.62	0.56
Butyl paraben	0.75	0.55	0.58	0.4	0.61	0.69	0.29
Creatinine standardized concentrations[a]							
No of sample pairs	28	25	29	29	21	22	26
2,4-dichlorophenol	0.88	0.65	0.63	0.69	0.41	0.41	0.22
2,5-dichlorophenol	0.77	0.89	0.71	0.66	0.44	0.81	0.38
Bisphenol A	0.22	0.05	0.07	0.19	0.38	0.53	-0.08
Benzophenone-3	0.85	0.85	0.71	0.73	0.82	0.65	0.47
Triclosan	0.64	0.68	0.54	0.48	0.74	0.56	0.49
Methyl paraben	0.60	0.61	0.57	0.51	0.58	0.65	0.58
Ethyl paraben	0.48	0.46	0.40	0.30	0.27	0.58	0.42
Propyl paraben	0.52	0.45	0.52	0.36	0.37	0.78	0.58
Butyl paraben	0.75	0.60	0.64	0.41	0.62	0.76	0.41
Crude concentrations							
No of sample pairs	32	32	30	36	24	29	30
2,4-dichlorophenol	0.67	0.75	0.40	0.58	0.43	0.29	0.16
2,5-dichlorophenol	0.84	0.64	0.52	0.57	0.49	0.40	0.32
Bisphenol A	0.43	0.14	0.48	0.41	0.36	0.22	0.06
Benzophenone-3	0.71	0.76	0.53	0.64	0.56	0.58	0.32
Triclosan	0.67	0.67	0.51	0.51	0.69	0.31	0.49
Methyl paraben	0.50	0.50	0.53	0.56	0.6	0.44	0.63
Ethyl paraben	0.46	0.54	0.41	0.44	0.57	0.51	0.35
Propyl paraben	0.57	0.32	0.64	0.45	0.55	0.49	0.51
Butyl paraben	0.72	0.63	0.63	0.49	0.49	0.64	0.28

[a] Creatinine concentrations were missing for 11 of the first spot urine samples.

Supplemental Material, Table S3: Adjusted[a] geometric mean (GM) of urinary phenol concentrations according to collection conditions among 213 urine samples from the 71 women of Study of Advanced Reproductive Age and Environmental Health, 2005 to 2008.

		2,4-DCP		2,5-DCP		BPA		BP3		TCS		MP		EP		PP		BP	
Collection Conditions	n	GM	p [b]	GM	p [b]	GM	p [b]	GM	p [b]	GM	p [b]	GM	p [b]	GM	p [b]	GM	p [b]	GM	p [b]
Gestational age (weeks)			0.39		0.49		0.01		0.49		0.3		0.44		0.87		0.25		0.64
≤ 18 [c]	54	0.7		12.2		1.0		105		15.8		159		4.1		38.3		1.8	
18.01 to 22	54	0.8		11.6		1.4		85.4		17.0		162		4.8		31.1		1.8	
22.01 to 32	58	0.9		13.8		1.3		71.5		15.6		123		3.9		22.8		1.4	
> 32	47	0.9		15.5		1.0		85.3		25.5		143		4.0		31.1		2.1	
Sampling season			0.87		0.33		0.3		0.02		0.98		0.17		0.30		0.1		0.31
Oct-March [c]	112	0.8		12.2		1.1		68.7		17.8		161		4.7		35.6		1.9	
April-Sep	101	0.8		14.2		1.3		110		17.9		130		3.7		25.1		1.5	
Hour of sampling			0.04		0.6		0.12		0.02		0.01		0.17		0.91		0.47		0.01
8.00 to 11.00 [c]	67	1.0		14.0		1.2		116		20.6		182		4.1		33.4		2.6	
11.01 to 13.00	56	0.9		13.2		1.0		56.8		25.0		136		4.7		35.1		1.9	
13.01 to 15.00	56	0.8		13.7		1.4		100		16.4		126		3.8		24.6		1.2	
15.01 to 18.00	34	0.6		10.5		1.2		71.8		8.8		132		4.2		26.8		1.2	
Day of sampling			0.69		0.45		0.28		0.81		0.95		0.56		0.20		0.75		0.38
Tuesday to Friday [c]	176	0.8		13.5		1.2		86.6		17.9		148		4.4		29.7		1.8	
Monday	37	0.8		11.6		1.0		81.8		17.6		133		3.1		32.2		1.4	
Specific gravity			<0.001		<0.001		<0.001		<0.001		<0.001		<0.001		0.11		<0.001		<0.001
1.002 to 1.009 [c]	74	0.4		5.4		0.6		54.5		10.1		69.7		3.3		13.5		1.0	
1.01 to 1.017	75	1.0		15.3		1.4		86.5		23.1		166		3.8		38.3		1.8	
1.018 to 1.036	64	1.8		30.5		2.2		143		25.3		293		6.0		57.8		3.1	

[a] Linear mixed models were simultaneously adjusted for the collection conditions and also for BMI, maternal age, year of sampling, maternal education and maternal race/ethnicity. [b] p-values of heterogeneity test. [c] Reference category.

Abbreviations: BP: butlyparaben, BPA: bisphenol A, BP3: benzophenone 3, DCP: dichlorophenol, EP: ethylparaben, MP: methylparaben, PP, propylparaben, TCS: triclosan
